# The Over-Feeding of Infants

**Published:** 1900-04-14

**Authors:** Eric Pritchard


### Hospital Clinics and Medical Progress.

### THE OVER-FEEDING OF INFANTS.

By Eric Pritchard, M.A., M.D. (Oxon).

The danger of over-feeding is not confined to artificially-fed infants, but applies, to a certain extent, to those brought up on the breast. With regard to the latter class, however, those that are nursed by their own mothers run comparatively small risk, because Nature, in a way, co-ordinates the mother's supply to the requirements of the offspring. Thus, a strong, healthy woman, with a full-sized, vigorous child, can generally supply it with a full allowance of milk; whereas the delicate, anæmic mother, with a correspondingly weakly baby, has probably a deficient mammary secretion—enough, however, for the infant's needs. There are, of course, exceptions to this rule, especially among the upper classes; but, speaking generally, the rule holds good. In the case of infants reared by a foster-mother the conditions are quite different; there is no natural co-ordination between the supply and the demand. When the services of a wet nurse are requisitioned it is generally because the child is premature, or in other ways delicate, this method of rearing being supposed to provide the best chance of success. If the child is strong and healthy, the less expensive, readier method of hand-feeding is oftener resorted to. In selecting a nurse for a delicate infant, the chief desideratum seems to be to find one who is buxom, healthy, and strong; in fact, one who presents the typical and traditional characteristics of a wet nurse. The weakly nurseling, thus provided, possibly thrives for a few days, but sooner or later the milk is said to disagree, and sickness and vomiting ensue. Another and another nurse, perhaps, is tried, but generally with equally unsatisfactory results, till finally recourse is had to hand-feeding, which, in spite of the initial handicap, will be successful if carried out on rational lines, and quantitatively and qualitatively adapted to the requirements of the infant. I believe that in very few cases in which wet nurses have proved a failure is it the quality of the milk which has been at fault. It is from first to last a matter of quantity. I have in several instances found that when the milk has been said to disagree, all symptoms of such disagreement have disappeared by drawing off the milk with a breast pump, and supplying in measured and appropriate quantities by means of a bottle. And further, I have had considerable success with premature and delicate children by selecting for them nurses who, though otherwise healthy, have been women of indifferent physique, with quantitatively a poor milk supply. In choosing a nurse for an infant, the main object in view should be to provide one whose physical standard bears a close relationship to that of the child; if one cannot be found to fulfil these conditions, the milk should be drawn off and given in measured quantities.

In the case of infants artificially reared the question of quantity is under much easier control, and consequently errors in this direction are less justifiable. But as a matter of fact, over-feeding in their case is of more frequent occurrence than in the case of breast-fed children. They are encouraged to take as much as possible, instead of having their sensitive digestive organs shielded by supplying them with no more than their actual requirements. It is particularly during the first few days of life that any excess in quantity is likely to do damage. The stomach at birth is relatively small, and only capable of containing something like an ounce of fluid; and though its capacity rapidly developes, it is of the utmost importance that its powers should not be overtaxed. Habits of vomiting—overflow the nurses call it—are easily engendered at this period, and, if not controlled, soon develop into serious complications. The following quantities may be regarded as the average amounts suited to the capacity of the infantile stomach during the successive stages of its development; variations may be desirable for particularly vigorous or feeble children, but for the infant of normal weight and size they are substantially correct:—

| Age of Infant. | 1 Day. | 2 Days. | 3—14 Days. | 14—28 Days. | 2 Months. | 3 Months. | 4—5 Months. | 6—7 Months. | 8—12 Months. |
|---|---|---|---|---|---|---|---|---|---|
| Quantity of food for each feeding ... ... ... | Oz. $\frac{1}{2}$ | Oz. $\frac{3}{4}$ | Oz. 1 | Oz. $1\frac{1}{2}$ | Oz. 3 | O . $3\frac{1}{2}$ | Oz. $4\frac{1}{2}$ | Oz. 6 | Oz. 8 |

On comparing these quantities with the usual amounts given to infants there will be found to be a great discrepancy. It is assumed that the food, as regards quality, is of the standard of human milk. If such milk be supplied to an infant in the above quantities its daily increment in weight and its general development will proceed with normal regularity, and no advantage will be gained by increasing the amounts. Although errors of over-feeding can generally be referred to excess in bulk, the mistake of giving the food in too concentrated a form frequently occurs, and the mistake most often made is that of adding some patent or prepared food to modified milk, which in itself is amply sufficient. These patent foods are the delight of mothers and nurses, they are so easily digested—in fact, they are already digested before they enter the infant's stomach—that they can be tolerated and assimilated in quantities which would be at once rejected if given in the form of milk, or other undigested food. The consequence is that the infant grows visibly fatter day by day, and to the inexperienced eye presents the appearance of an infant Hercules, veiling under its outward serenity the inevitable germs of future trouble. Under such conditions no mother can be induced to believe that her offspring is in anything but the most robust of health. Little straws of evidence, if inquired for, will, however, show which way the wind is blowing. The child, though fat and firm, will present an unduly open fontenelle; the bones of the head are soft; the muscular powers are weak, as evinced by its incapacity for holding up its head, and later on by sitting up and standing. It generally perspires a good deal, especially at the back of the neck; it is restless at night, and throws the clothes off; it does not like being lifted up or pulled about, and teething is late and troublesome. All these are the early symptoms of rickets, and in a condition of potential rickets the infant undoubtedly is. The mother is horrified if you tell her so, and does not believe you; but it is the case, nevertheless, although it may never reach the advanced stage of bandy legs and bent bones popularly associated with the word rickets. Such is the condition of the infant suffering from the first degree of over-feeding; and nearly all children fed on artificial foods, in addition to an adequate supply of diluted milk, will be found to conform to the above description. If the process of over-feeding reaches a more extreme stage, more obvious and serious symptoms associated with stomachic and intestinal troubles will supervene, and it is these troubles which are most constantly met with in the case of hand reared children. The symptoms are so numerous and varied that it is impossible to deal with them here, but they may be classified in two groups. Firstly, those which are due to the mechanical results of overfilling the stomach and intestines; in this group must be included the symptoms due to dilated stomach, which are largely nervous in character. The second group of symptoms comprises those which are due to the fermentative changes which occur in the excess of food in the stomach and intestine; these symptoms are the most serious of all; they begin with flatulence, colic, diarrhœa, and vomiting, and proceed to catarrhal inflammation of the bowel, wasting, convulsions, and the general atrophic condition known as marasmus. The vendors of patent foods have much to be responsible for in the false education of the mothers in matters of infant feeding: by literature, pictorial and otherwise, spread broadcast throughout the land they have become the chief medium for formulating the ideas of our young mothers as to their duties towards their infants in regard to feeding. To give an example of the mischievous instructions which they give for preparing an infant's daily food, I quote verbatim the directions as supplied with what is, perhaps, the best known of all patent foods; it certainly has an enormous sale, and has doubtless played no inconsiderable part in lowering the physical standard of the present generation in this country. The following are the instructions:—

"For children about the age of three months dissolve a tablespoonful of the food in four tablespoonsful of hot water, and add sufficient warm cow's milk to make half a pint," and, further, "the quantities mentioned in the directions will be found sufficient to fill a feeding bottle, and enough (*sic*) for a meal for an infant of three or four months. The food should be put in a feeding bottle, and the child allowed to suck until its contents are exhausted." It is not stated how many bottles should be given during the 24 hours, but presumably not less than eight for a child of three months. A simple calculation shows that if these directions are followed out, the infant will receive in the 24 hours 64 ozs. of raw milk, and eight tablespoonsful of the patent food. Sixty-four ounces of milk contain:—

| | |
|---|---|
| Fat ... ... ... ... | 2·240 oz. |
| Proteid ... ... ... | 2·56 „ |
| Sugar ... ... ... | 2·78 „ |

And eight tablespoonfuls (4 oz.) of the patent food (calculated according to the analysis supplied with the food) contains:—

| | |
|---|---|
| Fat ... ... ... ... | ·007 oz. |
| Proteid ... ... ... | ·4028 „ |
| Sugar ... ... ... | 2·72 „ |

The milk and patent food combined, giving a total of:—

| | |
|---|---|
| Fat ... ... ... ... | 2·247 oz. |
| Proteid ... ... ... | 2·962 „ |
| Sugar ... ... ... | 5·50 „ |

An infant's physiological requirements of the above constituents, according to Rotch, who is an accepted authority on this subject, are as follow:—

| | |
|---|---|
| Fat ... ... ... ... | 1·26 oz. |
| Proteid ... ... ... | ·63 „ |
| Sugar ... ... ... | 2·205 „ |

Comparing the quantities as required by physiological needs, and those recommended by the proprietors of the patent food, it will be noticed that, as regards gross bulk, the food is considerably more than two and a-half times that of the normal standard of requirements. The fat is twice the required amount, the sugar, or carbo-hydrate, two and a-half times, and the proteid more than four times too much. To make the comparison quite clear, I have arranged the figures in the form of a table side by side:—

| Table of quantities for infant three months old during 24 hours. | As recommended by proprietors of patent food. | Physiological requirements, Dr. Rotch. |
|---|---|---|
| Total quantity | 80 ounces | 31·5 ounces. |
| Total quantity of fat | 2·2472 ounces | 1·26 ounces. |
| Total quantity of sugar or carbohydrates | 5·5 ounces | 2·205 ounces. |
| Total quantity of proteid | 2·96 ounces | ·63 ounces. |
| Percentage composition | Fat 2·8 %<br>Carbohydrates 6·8 %<br>Proteid 3·7 % | Fat 4·0 %.<br>Carbohydrates 7·0 %<br>Proteid 2·0 %. |

An infant fed on a patent food in the above quantities is manifestly and grossly overfed. All patent foods are not, perhaps, directed to be given in such great excess, but all that I have examined err in the same direction, though not, perhaps, quite in the same degree. Patent foods no doubt have their legitimate uses, they can often be tolerated when other forms of food are rejected, but they should be prescribed in known and proper quantities, and only by those who have studied their chemical composition. They are quite as dangerous as medicines, and should only be handled by those qualified to deal with them.

